# Modeling Material Machining Conditions with Gear-Shaper Cutters with TiN_0.85_-Ti in Adhesive Wear Dominance Using Machine Learning Methods

**DOI:** 10.3390/ma17225567

**Published:** 2024-11-14

**Authors:** Maciej Kupczyk, Michał Leleń, Jerzy Józwik, Paweł Tomiło

**Affiliations:** 1 Institute of Mechanical Technology, Poznan University of Technology, 3 Piotrowo Street, 60-965 Poznan, Poland; maciej.kupczyk@put.poznan.pl; 2 Faculty of Mechanical Engineering, Lublin University of Technology, 36 Nadbystrzycka Street, 20-618 Lublin, Poland; j.jozwik@pollub.pl; 3 Faculty of Management, Lublin University of Technology, 38 Nadbystrzycka Street, 20-618 Lublin, Poland; p.tomilo@pollub.pl

**Keywords:** machining, carburizing steels, gear production, adhesive wear, titanium nitride coatings, tool durability, reactive pulse plasma method, Kolmogorov–Arnold Network, predictive modeling, cutting tools

## Abstract

This paper examines the challenges of machining structural alloy steels for carburizing, with a particular focus on gear manufacturing. TiN_0.85_-Ti coatings were applied to cutting tool blades to improve machining quality and tool life. The research, supported by mathematical modeling, demonstrated that these coatings significantly reduce adhesive wear and improve blade life. The Kolmogorov–Arnold Network (KAN) was identified as the most effective model comprehensively describing tool life as a function of cutting speed, coating thickness, and feed rate. The results indicate that gear production efficiency can be significantly increased using TiN_0.85_-Ti coatings.

## 1. Introduction

Research in the field of machine technology is very often joined by the emergence of new construction and tooling materials. The increasing use of construction materials with enhanced strength properties results from the constantly increasing demands on the operation of machinery and equipment. Gear components occupy a special place in this respect. In this aspect, steels with increased pressure resistance and the most favorable frictional and wear properties (for the conditions of tooth pair interaction) are used for gearwheels, as well as materials characterized by small deformations resulting from thermochemical treatment. The latter requirement is related to the global trend to make gears ready for machining. Steels of this type contain chromium and nickel as basic alloying additives [1,2,3,4,5] However, these materials pose significant problems in their machining process.

During gear cutting, especially of alloyed structural steels for carburizing, adhesion wear, manifested by the formation of workpiece material sticking on the cutting blades’ contact surfaces, occurs in addition to abrasive wear of the cutting blades [5,6,7,8,9,10,11]. This process results in damage to the tooth surfaces of the workpieces due to the adhesions formed. This dramatically reduces the service life of the cutting edge, which is determined by the technological quality criterion of the cut tooth (the surface roughness and accuracy of the cut tooth deteriorate) [5,12]. It has been found that the dimensions of the fractures increase with the increase of the cutting speed [5,12,13]. Results of tests carried out under laboratory and industrial conditions [5], as well as data from the literature [14,15,16,17], indicate an almost complete absence of sticking points on tool edges during circumferential toothing of the aforementioned steel grades at very low cutting speeds (below 0.08 m/s). The use of such low cutting speeds results in very low productivity when machining the gear teeth. Increasing the machining productivity of these steels while maintaining the technological quality requirements of the toothing therefore becomes an important production problem, given the increasing use of this type of steel for heavy-duty toothed components [5].

It should be noted that when the build-up reaches an excessive size, it makes sharpening the tool lengthy and very expensive. In extreme cases, the modular chisel (also called gear-shaper cutter) may not be suitable for reconditioning. Sometimes, honing or stoning the flank faces of the modular chisel blades is attempted to remove the build-up. However, this is not always effective, as it can introduce significant errors in the tooth profile when cutting with a tool reconditioned in this way [5]. Therefore, the only reliable way to remove the build-up zone is to grind the rake face until the newly formed cutting edge is above the area with sticking (build-up made of the workpiece material).

In connection with the selection of the structural carburizing alloy steel 16HG) [18] for testing, an attempt was made in earlier studies [4] to interpret the formation of build-up and to formulate premises for preventing the dominance of adhesive wear in the process of cutting tool wear in HS6-5-2 (formerly SW7M) steel. Based on, among other things, the assumptions resulting from the configurational solid model [19], an attempt was made to prevent or partly eliminate the dominance of adhesive wear (manifested by build-up formation) by forming the surface layer of the cutting tool in a way that ensures a possibly low chemical affinity between the workpiece material (carburizing alloy structural steel 16HG) and the tool cutting material (high-speed steel HS6-5-2). Consideration was also given to the need to increase the wear resistance of cutting blades by increasing the hardness of their surface layer. To this end, work was undertaken to determine the suitability of using hard coatings with low chemical affinity to iron applied to the working surfaces of HS6-5-2 high-speed steel modular chisels [19].

On the basis of research [5,19,20,21,22], a modified titanium nitride coating with a higher titanium content in relation to the stoichiometric composition was selected for use in impact operation. The developed TiN_x_-Ti coating (where x = 0.85) is characterized by increased fracture toughness due to its chemical composition [19]. This composite coating material (a mixture of titanium nitride and a solid nitrogen solution in titanium [20,21,22]) was produced using a physical vapor deposition technique at a relatively low temperature to which the substrate (tool) is heated during coating deposition (500–600 K—approximately 230–330 °C). The low temperature does not affect the structure of the high-speed steel tool or its dimensional tolerances. Specifically, in this case, the RPP (Reactive-Pulse-Plasma) method, which belongs to the PVD (Physical Vapor Deposition) technique, was used to produce the antiwear coating [5,19,20,21,22]. The TiN_0.85_-Ti coating with a hardness of about 1450 HV, due to its low chemical affinity to iron, eliminated the adhesive wear of the modular chisel blades, thus improving the quality of the cut gears [5]. During this study, only abrasive wear of the blades was observed.

Having solved the problem of eliminating adhesion wear on blades when machining steels for carburizing, a comprehensive determination remained a significant problem:

- The optimum machining parameter area for tools coated with TiN_0.85_-Ti under new machining conditions, i.e., under predominantly abrasive wear;
- Determination of the thickness of the modified titanium nitride coating that is most favorable in terms of the durability of cutting blades used in impact work.

Mathematical modeling was used to comprehensively describe the circumferential chiseling process to determine the optimum machining parameter area and the most coating thickness. Both linear and non-linear models will be included in the studies to provide a comprehensive understanding of their capabilities and limitations for a definition of average modular chisel durability based on cutting velocity *v_c_*, coating thickness *g*, and circumferential feed *f_s_*. Therefore, the novelty of this work includes the indication of the mathematical model that best describes the modular chisel process and the statement of the suitability of the TiN_0.85_-Ti composite coating under impact work conditions. This study introduces also a novel technique of manufacture and application of TiN0.85-Ti coatings to gear manufacturing processes, specifically for carburizing alloy steels, addressing a critical challenge in reducing adhesive wear and extending tool life. By employing the Kolmogorov–Arnold Network (KAN) for predictive modeling, this research identifies optimal machining parameters—cutting speed, coating thickness, and feed rate necessary for enhanced tool durability and process efficiency. These findings fill a significant gap in existing knowledge regarding using high-speed steel tools in heavy-duty gear production under abrasive wear conditions, providing actionable guidelines for improving productivity and quality in industrial machining applications.

## 2. Materials and Methods

### 2.1. Research Stands

For the coating of modular chisels with TiN_0.85_-Ti coating, a device with an original design and operating principle was used, whose creators were a team of employees of the Institute of Materials Engineering of the Warsaw University of Technology [20,21,22].

**Figure 1:**
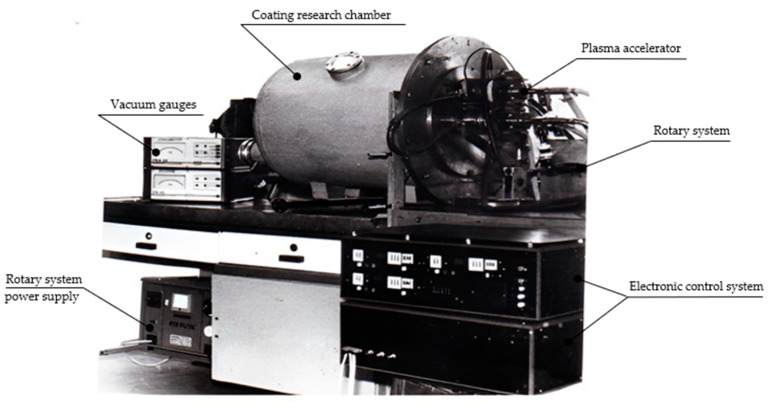
General view of the Reactive-Pulse-Plasma (RPP) hard infusible coatings manufacturing bench developed at the Warsaw University of Technology.

A general view of the Reactive-Pulse-Plasma (RPP) hard infusible coatings manufacturing is shown in Figure 1.

The essence of this method is the use of electrical pulse discharges for plasma generation, resulting in the generation of highly ionized and non-isothermal plasma. The advantages of the pulsed plasma process are its ease of operation and control, the simplicity of the device design, and its versatility.

The primary component of the device is a coaxial pulsed plasma generator connected to a vacuum coating deposition chamber. The pulsed plasma generator consists of two coaxial electrodes between which a strong pulsed electrical discharge is generated under reduced pressure. The outer electrode of the generator is extensively water-cooled and has a purely electrical function. The central electrode is heated by the Joule heat given off during the current flow and by the friction of the plasma “disk” moving along it [20,21,22].

As a result of the pulsed heating of the electrode, the surface of the electrode is vaporized, and these vapors become plasma components [20,21,22]. The second source of plasma components is gas introduced into the space between the electrodes. The gases are supplied from cylinders via pressure regulators. The amount of gas fed into the chamber is regulated by needle valves.

The pulsed electrical discharge in the interelectrode space is obtained by discharging a capacitor bank connected to the discharge circuit via a switch (ignitron). The ignitron is controlled by an electronic circuit that allows the frequency of the pulse trigger to be adjusted and the number of pulses to be controlled. The 200 µF capacitor battery is powered by a DC power supply with an adjustable output voltage in the range of 0 kV to 10 kV [20,21,22].

The tools on which the coatings are deposited can be in a plane that is freely inclined to the generator axis (from 0° to 90°) and can rotate around an axis perpendicular to the direction of plasma outflow from the generator (this is made possible by a specially designed holder). The coatings were produced using a discharge voltage of 4.5 kV. The capacitor battery was discharged every 2 s.

It should be noted, however, that before the coatings were deposited, the substrate surfaces (modular chisels) were subjected to a complex preparatory procedure. It consisted of two stages: (1) preliminary cleaning, and (2) final cleaning. The preliminary cleaning consisted of first cleaning the surface with a non-woven fabric (removing burrs) and then polishing with a suspension with diamond grains of dimensions from 1 to 9 μm. Then the chisel surfaces were cleaned with white spirit and rinsed in acetone. After these activities, the tools were rinsed in deionized water in the presence of ultrasound and then dried. The final cleaning consisted of cathodic etching with the tools as the cathode. The basic parameters of this process include etching time of 10 min, gas shield-argon, a pressure of 3 *×* 10^−3^ Pa, and voltage of 1500 V.

### 2.2. Tools Used in Research

Modular chisels with the symbol NNMb 3/200B, z = 34, made of HS6-5-2 (SW7M) steel with the number of blades *z =* 34, module *m =* 3 mm, and nominal pitch diameter *d*_o *nom*_ = 100 mm were used in the tests. The nominal pitch diameter was measured with an error range of ±0.2 mm. These chisels, prior to application of the TiN_x_-Ti coating (where x = 0.85), were checked according to a preset numerical program on an EFMS 630-type (Höfler, Malsch, Baden-Württemberg, Germany). involute meter, adapted, among other things, to measuring the outline of the revolver and the tooth inclination line (tooth line inclination angle), and on a CAT 85 instrument consisting of instruments for measuring the circumferential pitch, tooth run-out, and units-control, counting, and estimating measurement results. The measurement of the tooth inclination angle was conducted with an error margin of ±0.5°. Both instruments (EFMS evanescent meter and CAT 85) are computer-assisted devices (Höfler, Malsch, Baden-Württemberg, Germany). Deviation measurements on the CAT 85 device and the EFMS 630 involute meter are performed with an accuracy of 0.1 μm. The measurement is automatic. The results are obtained in the form of a ready printout of the deviation values of the measured quantities.

As there is no data in the PN on the accuracy of the manufacturing of modular chisels, the recommendations given in [23], were used to assess their accuracy. Chisels that did not comply with the above-mentioned standards were not qualified for in-service testing.

**Figure 2:**
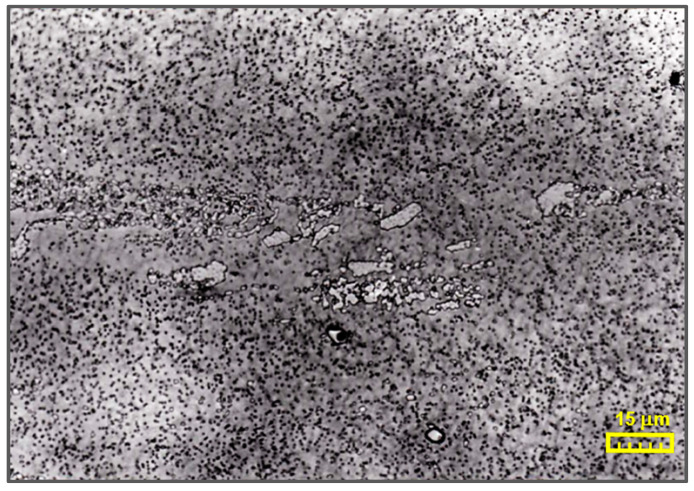
Image of HS6-5-2 steel structure (etched with 10% HNO_3_ solution).

In order to eliminate differences in physicochemical properties and their influence on the test results, all chisels were subjected to hardness tests and chemical composition tests prior to cutting the gear teeth, with comparison with the requirements of [24] for the finished product in the hardened and tempered state (Table 1). Hardness values were obtained with a measurement error of ±1 HRC. An image of the HS6-5-2 steel structure is shown in Figure 2. The Fischerscope X-ray XDV-SDD Fisher X-ray fluorescence spectrometer (Helmut Fisher GmbH, Sindelfingen near Stuttgart, Germany), was used to assess the chemical composition. It is a universal device for non-destructive, precise, easy-to-use measurement of chemical composition (X-ray fluorescence microanalysis) from a small area. It is used to assess the composition of bulk materials and thin coatings and to precisely measure the thickness of coatings with the possibility of computer processing of test results, their graphic visualization and archiving, and microscopic observation. It is equipped with a video system.

Hardness measurements were taken at four locations on the so-called thrust surface of the modular chisel. The hardness measurement of modular chisels was performed on an automatic Rockwell hardness tester type KP15002 “KABID-PRESS” by Research Equipment Production Company “Press” (also known as “KABID-PRESS”) Warsaw, Poland. The permissible error of indication is ±1 HRC; the diamond indenter was loaded with a force of 1470 N.

The geometry of the blades was also verified according to the recommendations given in [23,24], as well as the roughness of the lateral application surfaces. The roughness measurement was performed on a profilograph-profilometer type A1 model 252 manufactured by the “Kalibr” plant, using a recording with an RC filter. This filter allows for the elimination of the curvature (involute outline) of the blade. The measurements were performed at a sensor feed speed of 6 mm/min.

Particular attention was paid to the condition of the cutting edges. Sharpening of the chisels was carried out in such a way as to prevent grinding burns. A T1-250x32x76-99A-46-K-5-V, is produced by the company Tyrolit, headquartered in Schwaz, Austria, grinding wheel was used at 2700 rpm; the chisel speed was 230 rpm-counter-rotating grinding (the grinding wheel dimensions were chosen to match the geometry of the chisels being sharpened).

After being coated with TiN_x_-Ti (where x = 0.85), the modular chisels were subjected to several tests to ascertain their assumed performance characteristics as well as the quality of the coating itself. The identified parameters and quality characteristics of the coating and modular chisels after coating included coating adhesion to the substrate, coating microhardness, coating, roughness of the coated chisel’s evolute surface, radius of the rounded cutting edge, and coating thickness.

The microhardness of the coatings was measured on the apical surfaces of the blades at a load of 0.5 N. The load used was in accordance with the recommendations of [25]. The load applied is in accordance with the recommendations of [25]. Microhardness tests were performed on a Vickers hardness tester, PICODENTOR HM500 (producent Helmut Fisher GmbH, Sindelfingen near Stuttgart, Germany)The device is equipped with a programable XY table with a positioning accuracy of ≤0.5 μm. In order to minimize the substrate effect on the coating microhardness result, hardness measurements were performed for coatings with a thickness of 7 μm. The representative value was the average of 9 measurements. The measurements showed coating hardness in the range of 14–15 GPa. The tests carried out showed that the coatings produced by the RPP method with the adopted chemical composition have a significantly lower hardness than titanium nitride coatings with stoichiometric composition. The good performance of these coatings under impact operating conditions, despite their lower hardness, can be explained by their much higher resistance to brittle fracture (tested using the Palmqvist method [26,27]) and their very finely dispersed, compact structure [20,21,22], as well as the pulsed nature of the coating application to the substrate. Processes with the continuous nature of coating application often produce a fibrous structure, sometimes with high porosity, which greatly reduces the resistance of these coatings to mechanical wear [20,21,22].

The rounding radius of the cutting edge after the coating process averaged about 0.025 mm and was greater than that of uncoated tools (by about 0.015 mm). The radius of roundness of the cutting edge no greater than 0.03 mm was used as a criterion for accepting a coated tool for use.

The tools were coated with TiN_0.85_-Ti coatings of three thicknesses *g_i_*_(*i* = 1, 2, 3)_: 1 μm, 4 μm, and 7 μm. These values correspond to the average thicknesses of the coatings on the flank faces and are applied to four randomly selected blades of each modular chisel. An instrument adapted for measuring non-magnetic coatings on magnetic substrates was used to measure the thickness of titanium nitride coatings. The DELTASCOPE MP2-GA2H layer gauge (producent Helmut Fisher GmbH, Sindelfingen near Stuttgart Germany) allows measurements with an accuracy of ±0.1 μm in the range 0–1.2 μm for a minimum measurement area *φ* of 1.5 mm and a minimum radius of curvature of 1.5 mm. This instrument operates on the magneto-inductive principle in accordance with [28].The thickness measurements of the TiN_0.85_-Ti coating were performed on the flanked faces of four randomly selected modular chisel blades.

Adhesion tests based on the evaluation of the level of the acoustic emission signal and the level of the vibration signal, measured by the level of the amplitude of the vibration acceleration in the tested frequency band, were performed using an automatic, computer-aided scratch test device [29]. This device was equipped with two measurement paths to determine the vibration and acoustic signal.

The following parameters were adopted for the scratch test:

- Drawing speed *dx*/*dt* = 7.5 mm/min,
- Loading rate *dL*/*dt* = 300 N/min,
- Ratio of loading rate to drawing speed *dL/dx* = 40 N/mm,
- Tip radius of the stylus (Rockwell indenter) *R* = 0.2 mm.

The TiN_0.85_-Ti coating was considered sufficiently adhesive if the critical load value *P_kr_* was no less than 40 N on four randomly selected chisel blades.

After being positively verified, the tools subjected to the above tests were put into service (all tests were non-destructive).

In addition, it should be mentioned that the titanium nitride coatings (TiN_0.85_-Ti) produced with the modified composition were silver in color due to the predominance of titanium as compared to titanium nitride coatings with stoichiometric composition, which have a gold color [20,21,22]. The work uses advanced modeling methods based on machine learning [30,31,32,33], taking into account a range of physical phenomena, including those described in the [34,35,36,37,38,39,40,41,42,43,44].

### 2.3. Workpiece Material Used in the Tests

The choice of structural alloy steel for carburizing, 16HG (PN/EN 10084, ISO/EN 1.7131), for the tests was primarily dictated by the following:

- The worldwide trend towards the use of materials for gears (gear teeth), which are characterized by a few-dimensional and shape changes as possible after thermo-chemical treatment, due to the manufacture of the gear teeth components ready for this treatment;
- The occurrence of predominant adhesive wear of uncoated modular chisel blades when cutting gear teeth from this steel grade;
- In own research conducted under industrial conditions [5,12,19];
- The increasing use of this material (16HG steel) for toothed components [1,2,3,4,5];

The starting semi-finished products were certified shafts with a diameter of *ϕ* 120 mm and a length of *l* = 3000 mm.

In order to eliminate the differences in the physical and chemical properties and their influence on the test results, samples of the workpiece material were taken in accordance with [45] to test the mechanical properties, and the correctness of the chemical composition was determined on the basis of [46] (Table 2).

**Figure 3:**
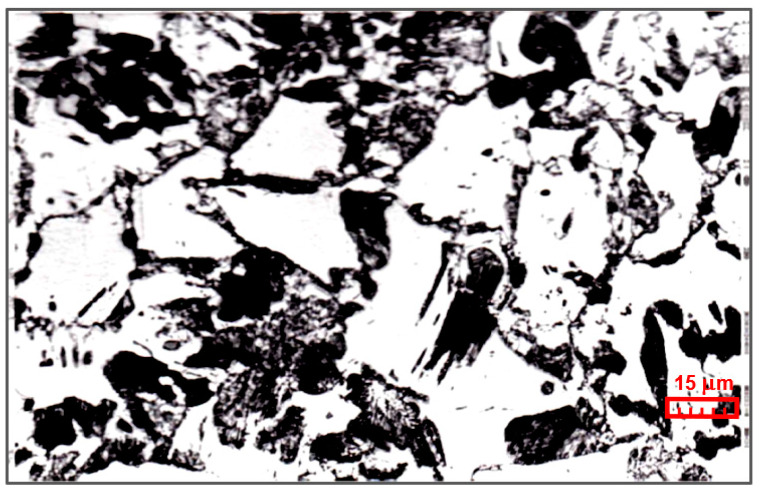
Image of the 16HG steel structure.

The workpiece material was in a softened state. Figure 3 shows an image of the structure of the 16HG steel.

In order to check the mechanical properties, samples were prepared in accordance with the requirements of [47,48]. The results (average of four measurements) of the mechanical properties are presented in Table 3.

It should be noted that the strength properties and impact strength of the qualification specimens were checked in accordance with EN 10083-1+A1:1999 in the heat-treated state (quenching 1130 K-oil, tempering 450 K-air), and the hardness in the softened state.

After checking the chemical composition and mechanical properties, the qualified shafts were cut into discs, which were used to make the cylindrical-shaped blank for cutting the gear teeth. The cylindrical-shaped blanks had a top diameter of ϕ114_−0.05_ mm, a pinhole diameter of ϕ30H7 (30^+0.021^), and a rim width of 32 ± 0.1 mm.

All cylindrical-shaped blanks were checked for hardness by measuring at four points on both faces. The hardness determined the final qualification of the surround for testing. Blanks with a hardness of 180 ± 5 HB were qualified for testing.

The hardness measurement of the workpiece material was performed on an automatic Brinell hardness tester type KP15002 “KABID-PRESS” Warsaw with a loading force of 7355 N and a ball diameter of ϕ5 mm. Diagonal measurements for Brinell hardness evaluation were performed with a resolution of 0.001 mm.

### 2.4. Description of the Machining Station (Rotary Burr Chaser)

Gear cutting of straight helical gears was carried out on an OHA 50A-type rotary gear cutting machine with automatic cycle, manufactured by TOS Čelakovice (manufacturer of machine tools based in Čelákovice, Czech Republic), with the following most relevant technical data:

- Achievable toothing chiseling accuracy-grade 6 according to DIN 3962;
- Number of double feeds per cycle-infinitely adjustable from 45 to 720 2·pitch/min;
- Peripheral feed rate-infinitely adjustable from 0.01 to 0.55 mm/2·pitch;
- Max. diameter of gear tips-*ϕ*500 mm;
- Min. diameter of gear tips-*ϕ*50 mm;
- Max. gear module-8 mm;
- Smallest number of teeth of the chiseled gear-*z* = 12.

Before cutting the serrations, the chisel was checked for accuracy in accordance with the requirements of [49].

## 3. Results

Table 4 shows the results of tests on the tool life of modular chisel blades without and with TiN_0.85_-Ti coating as a function of peripheral feed *f_s_* for the respective tested cutting speeds (*v_c_*_1_ = 0.16 mm/s, *v_c_*_2_ = 0.25 mm/s and *v_c_*_3_ = 0.33 mm/s) and blunt index *VB_B_*_max_ = 0.20 mm when machining serrations from 16HG steel.

**Figure 4:**
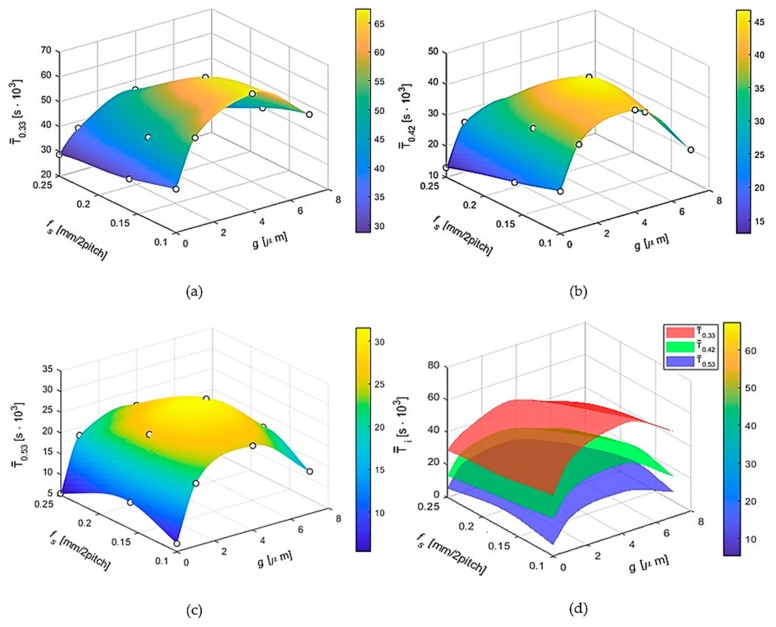
The course of the variation of durability $\bar{T}$*_vci_* of modular chisel blades as a function of coating thickness *g* and peripheral feed rate *f_s_* for different cutting speeds: (**a**) *v_c_*_1_ = 0.33 m/s, (**b**) *v_c_*_1_ = 0.42 m/s, (**c**) *v_c_*_1_ = 0.53 m/s, and (**d**) comparative summary of test results showing the effect of coating thickness *g* and feed rate *f_s_* for the adopted cutting speed values *v_ci_*, on the durability of modular chisel blades obtained from Surface Plot $\bar{T}$*_vci_* models with function Cubic Spline Interpolation.

The graphs in Figure 4 show the dependence of the tool life of the modular chisel blades (without and with TiN_0.85_-Ti coating) on the coating thickness *g* and the peripheral feed rate *f_s_* for the different adopted cutting speeds *v_c_*_1_, *v_c_*_2_, and *v_c_*_3_. In the following figures, all combinations of the above-mentioned parameters are taken into account.

For the present graphs, the cubic spline interpolation method was used. This is an approximation method that produces smooth curves from a set of data points. Cubic spline interpolation uses third-order polynomials between data points, resulting in smooth transitions between them. A cubic spline between two points can be described by the following equation:

*S_i_*(*x*) = *a_i_* + *b_i_* (*x* − *x_i_*) + *c_i_*(*x* − *x_i_*)*^2^* + *d_i_*(*x − xi*)*^3^* (1)

where:

*S_i_*(*x*)-spline function for the interval *i*

*x_i_*-nodes (data points).

*a_i_*, *b_i_*, *c_i_*, *d_i_*-coefficients that are determined by smoothness and continuity conditions.

Figure 4 shows a graphical interpretation of the change in tool life $\bar{T}$_0.33_ of modular chisel blades as a function of coating thickness *g* and peripheral feed *f_s_* for cutting speed *v_c_*_1_ = 0.33 m/s. The tool life values shown in the graphs were determined for a blunting index *VB_Bmax_* = 0.20 mm.

The graph in Figure 4a shows that blade life increases as coating thickness increases between 1 μm and 4 μm and decreases between 4 μm and 7 μm. In all cases, however, the durability of coated modular chisels is higher than that of uncoated tools. In Figure 4, a gradual decrease in the life of the modular chisel blades can also be observed as the value of the peripheral feed *f_s_* increases for the applied cutting speed of *v_c_*_1_ = 0.33 m/s.

Figure 4b shows a graph showing the dependence of the life $\bar{T}$_0.42_ of the cutting blades as a function of the coating thickness *g* and the peripheral feed *f_s_* for a cutting speed *v_c_*_2_ = 0.42 m/s. The graph in Figure 4b for *v_c_*_2_ = 0.42 m/s shows, similarly to the graph for a cutting speed of 0.33 m/s, that an increase in blade life occurs as the coating thickness increases in the range from 1 μm to 4 μm and a decrease in the range from 4 μm to 7 μm. This graph shows, similar to Figure 4a, that there is a gradual decrease in the durability of the modular chisel blades as the value of the peripheral feed rate *f_s_* increases for the applied cutting speed of *v_c_*_2_ = 0.42 m/s.

Figure 4c provides a graph showing modular chisel blade life’s dependence on cutting speed *v_c_*_3_ = 0.53 m/s on titanium nitride coating thickness and peripheral feed rate. This graph shows that the tools have the highest tool life for both the intermediate applied peripheral feed rate *f_s_*_2_ = 0.16 mm/2·pitch and the adopted intermediate TiN_0.85_-Ti coating thickness of *g*_2_ = 4 μm.

Figure 4d presents comparative plots showing the effect of the parameters studied, i.e., coating thickness *g*, feed *f_s_* for the adopted cutting speeds *v_ci_*, on the durability of the modular chisel blades. All data were approximated using cubic spline interpolation to produce smooth surfaces that match the actual data points.

The graphs in Figure 4d show that, as the value of the cutting speed increases, blade life decreases for all types of tools tested (without and with an antiwear coating).

**Figure 5:**
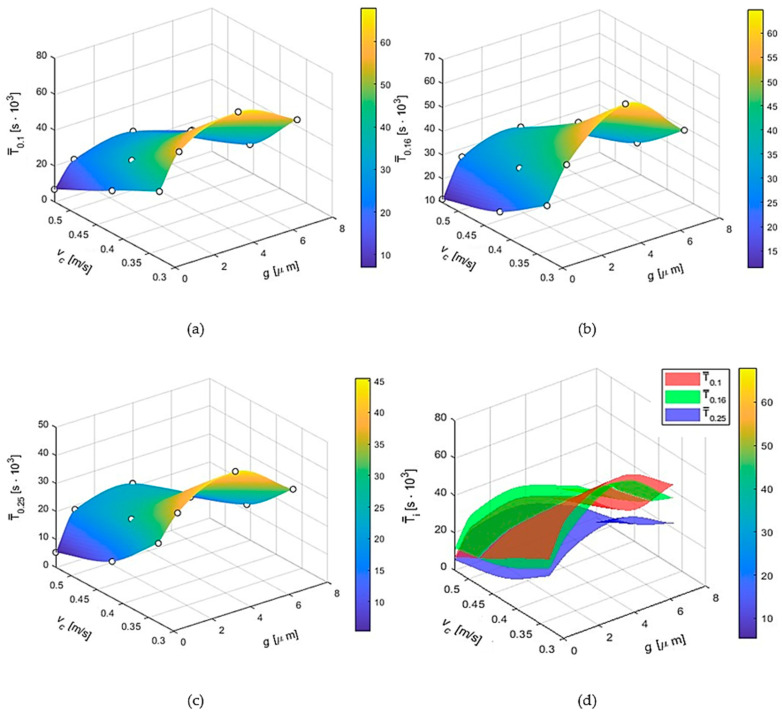
The course of variation in the life $\bar{T}$*_fsi_* of modular chisel blades for a circumferential feed *f_si_* as a function of coating thickness *g* and cutting speed *v_c_*, obtained from the Surface Plot interpolation models using the Cubic Spline Interpolation function, for different circumferential feeds: (**a**) *f_s_*_1_ = 0.10 mm/2·pitch, (**b**) *f_s_*_2_ = 0.16 mm/2·pitch, (**c**) *f_s_*_3_ = 0.25 mm/2·pitch, and (**d**) comparative summary of test results showing the effect of coating thickness *g* and cutting speed *v_c_* for individual peripheral feed rate values (*f*_si_) on the durability of modular chisels, obtained from the Surface Plot interpolation model $\bar{T}$*_fsi_* with Cubic Spline Interpolation functions.

Figure 5 graphically show the dependence of the tool life of the modular chisel blades during the machining of wheels made of 16HG steel for the respective applied values of peripheral feed rate *f_s_* as a function of the cutting speed *v_c_* and the thickness *g* of the titanium nitride coating and for the uncoated tool (*g* = 0 μm). The tool life values shown in the graphs were determined for the blunt index *VB_Bmax_* = 0.20 mm.

Figure 5a provides a graph showing the effect of TiN_0.85_-Ti coating thickness and cutting speed on the tool life of the tools tested for the smallest peripheral feed rate used, *f_s_*_1_ = 0.10 mm/2·pitch. The graph in Figure 5a shows that as the cutting speed increases, the durability of modular chisels decreases, while the highest durability is observed for titanium nitride-coated tools with an intermediate applied coating thickness of 4 μm.

Figure 5b shows a graph of the variation in tool life of modular chisels (without and with TiN_0.85_-Ti coating) as a function of cutting speed *v_c_* and titanium nitride coating thickness for an intermediate applied peripheral feed rate *f_s_*_2_ = 0.16 mm/2·pitch.

From the graph presented above (Figure 5b), it can be seen that the highest tool life $\bar{T}$_0.16_ in machining 16HG steel for peripheral feed rate *f_s_*_2_ = 0.16 mm/2·pitch is shown by modular chisels for the lowest cutting speed used. Their tool life decreases with increasing cutting speed. The influence of the thickness of the titanium nitride coating (TiN_0.85_-Ti) on durability is more complex. As in the previously considered cases, tools with a coating with an intermediate applied thickness of *g*_2_ = 4 μm have the highest durability. However, in each case, regardless of the thickness of the applied coating (1 μm, 4 μm, 7 μm), the durability of the coated tools was significantly higher than the uncoated ones.

Figure 5c shows a graph showing the dependence of the life of modular chisels when cutting serrations in 16HG steel on coating thickness *g* and cutting speed *v_c_* for the highest peripheral feed rate applied *f_s3_* = 0.25 mm/2·pitch. It can be seen from Figure 5c (as well as from the graphs in Figure 5a,b), that the tool life of cutting tool blades for peripheral feed rate *f_s_* = 0.25 mm/2·pitch decreases with increasing cutting speed. Again, modular chisels with a coating of *g*_2_ = 4 μm have the highest tool life. The lowest tool life is that of tools without an antiwear coating.

Figure 5d shows a comparative summary of graphs showing the effects of the tested parameters, i.e., coating thickness g and cutting speed vc for the individual peripheral feed rate values used in this study (*f_s_*_1_ = 0.10 mm/2·pitch, *f_s_*_2_ = 0.16 mm/2·pitch and *f_s_*_3_ = 0.25 mm/2·pitch) on the durability of the modular chisels. All three graphs were generated using the cubic spline interpolation method. This is an approximation technique that uses third-order polynomial functions to create smooth surfaces from the available data.

## 4. Modeling Results

**Figure 6:**
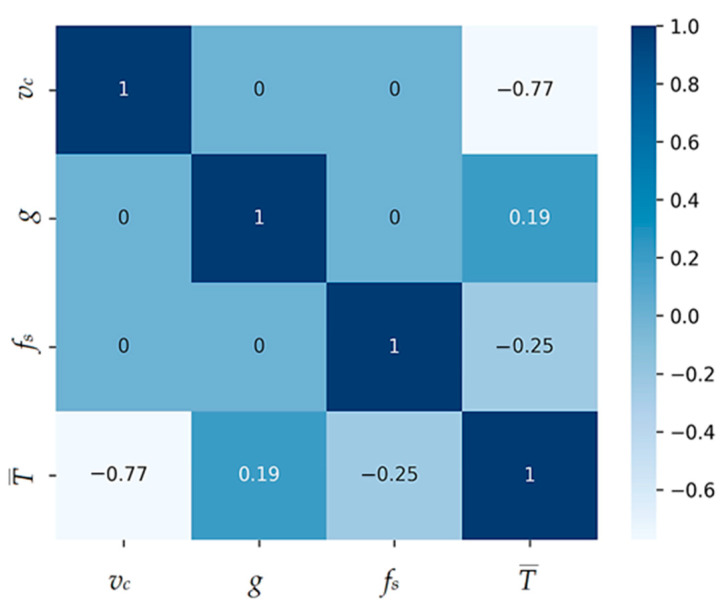
Sperman correlation matrix of model parameters.

Both linear and non-linear models will be included in this study to provide a comprehensive understanding of their capabilities and limitations for predicting average chisel durability based on cutting velocity *v_c_*, coating thickness *g,* and peripheral feed rate f. Two models will be created, one to determine the average chisel life $\bar{T}$, and the other to determine the chisel life in phase i for values from the set $\left\{T_{I},T_{2},T_{3},T_{4},T_{5}\right\}$ where *T_i_* _(i = 1, 2, 3, 4, 5)_ means the durability of individual tools from which the average durability value was determined. To examine the correlation between the variables for the first model, a correlation matrix was created based on the Spearman correlation coefficient due to the data’s lack of normality of distribution. The correlation matrix for the first model is shown in Figure 6.

The correlation matrix shows that the output value from the $\bar{T}$ model is negatively correlated with *v_c_*, i.e., as the cutting speed increases, blade life decreases significantly. There is a weak correlation between the $f_{s}$ and g parameters. This is confirmed by the graphs in Figure 4 and Figure 5, where a non-uniform (variable) effect of feed rate and coating thickness as their values increase on blade life can be observed. The input parameters $v_{c,}$ g and $f_{s}$ are not correlated with each other. In order to select the optimal model to describe the influence of the analyzed parameters on the durability of the modular chisel blades, the following were considered:

- LinearRegression;
- DecisionTreeRegressor;
- GradientBoostingRegressor [30];
- AdaBoostRegressor [31];
- Kolmogorov–Arnold Network (KAN) [32].

The first step of the model development process was to normalize the values to an interval from 0 to 1 by applying scaling against the maximum value. In the rest of the article, parameter designations without units refer to normalized values.

**Figure 7:**
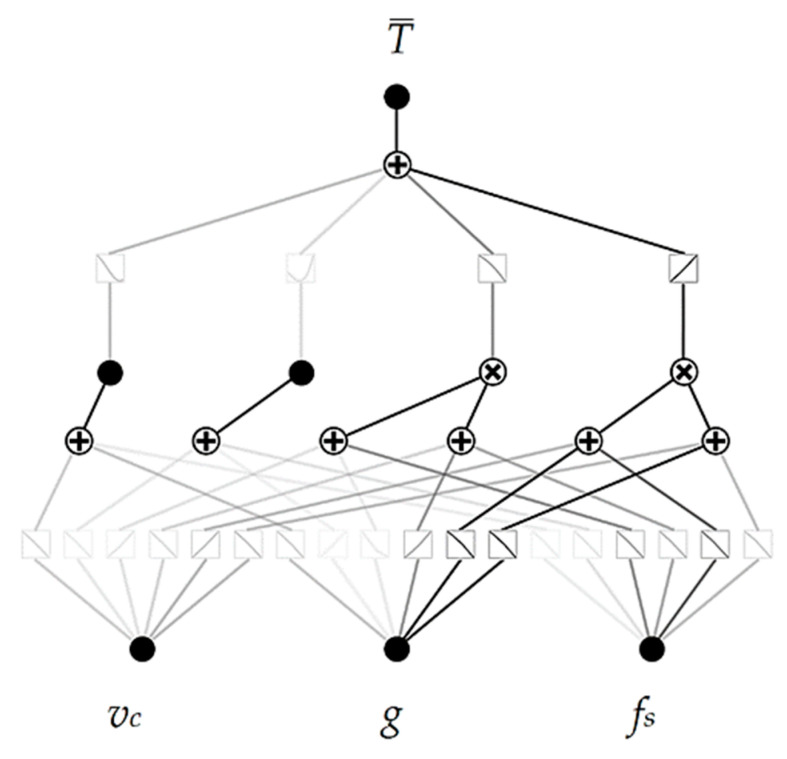
The base model architecture of the developed KAN model.

In each case, model parameters were chosen to avoid overfitting with low complexity and high estimation accuracy. The DecisionTreeRegressor was initialized with a maximum depth of 4. The decision tree’s depth determines the complexity level with which the model will segment the data into groups. In the case of modeled data where there are 3 variables in the input and 1 in the output, a depth of 4 can provide good enough performance without overcomplication and without the occurrence of overfitting. The gradient boosting regressor was initialized with a number of estimators of 20 at which boosting is terminated. In addition, an early stopping mechanism is used in case a perfect fit occurs. The AdaBoostRegressor was initialized with a maximum depth of 3 (default value) and a maximum number of estimators of 100. Again, the parameters were chosen because of the trade-off between model complexity and accuracy. The base-developed KAN model architecture consisted of one layer using 2 summation nodes and 2 multiplication nodes, one hidden neuron was used, and approximation using cubic spline and grid intervals of 5 [31,32]. The piecewise segments of the learnable spline activation functions in KANs are referred to as grid intervals. The value of 5 was chosen because the density of the grid is quite good while maintaining a lower degree of complexity, which in a further stage will reduce the error when approximating the model by equation. The base model architecture of the developed KAN model is shown in Figure 7.

**Figure 8:**
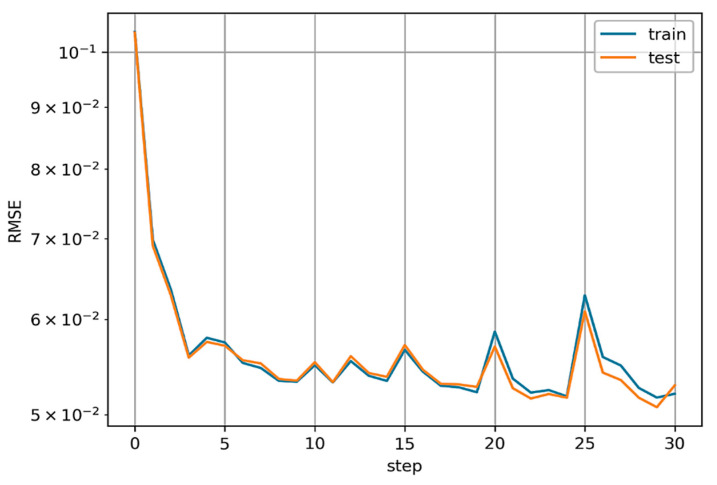
The course of the loss function values RMSE.

The KAN model was trained for a target number of steps (pass through the entire data set) of 30, using the LBFGS (limited-memory Broyden Fletcher Goldfarb Shanno) optimizer. LBFGS is an optimizer belonging to quasi-Newtonian methods, so it is responsible for approximating the second derivative of the loss function (the Hessian matrix) without directly calculating it. The optimal values of the loss function were obtained at step 24. The course of the loss function values in the form of RMSE is shown in Figure 8.

**Figure 9:**
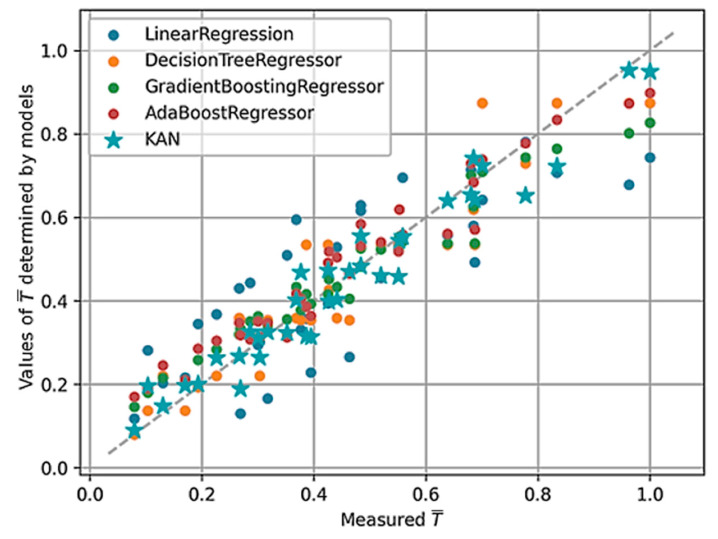
Comparison of values of T determined by models with measured values.

The models were compared using metrics in the form of Coefficient of Determination (R^2^), Mean Absolute Error (MSE), and Root Mean Square Error (RMSE). The range of variability of the R^2^-score is from 0 to 1, where 0 means no predictive value and 1 indicates the model perfectly predicts values. MSE and RMSE are calculated based on the mean value, so their range of variation depends on the analyzed data. The lower the value of these coefficients, the smaller the error. Of the models developed, the best metrics fell to the KAN model, which achieved an R^2^-score of 0.945 with an MAE of 0.04 and an RMSE of 0.05. A comparison of the models’ metrics is shown in Table 5, while Figure 9 shows a comparison of determined based on the model with measured values.

**Figure 10:**
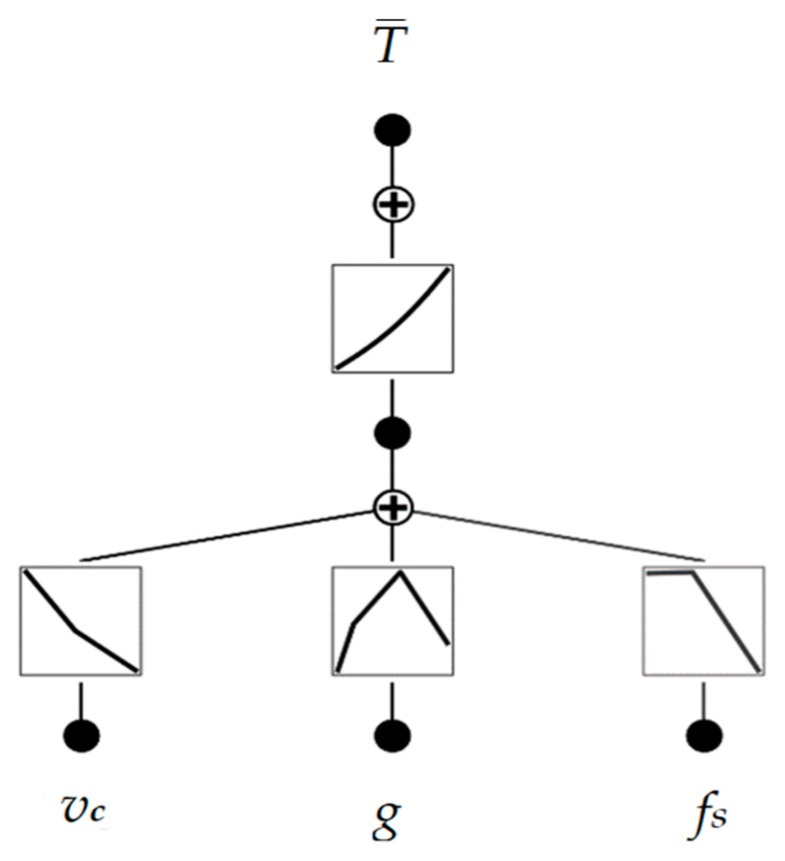
Architecture of the KAN model after simplification.

The advantage of using the KAN model is that they can be interpreted, and the complexity is reduced by using a pruning operation. The pruning operation is based on the identification of connections and neurons whose weights are close to 0 or whose influence on the final result is small. The identified element is removed from the model architecture. This operation reduces the number of nodes in the architecture, which facilitates interpretability. The architecture of the model after the pruning operation is shown in Figure 10.

After the pruning procedure is completed, it is possible to obtain the regression Equation (2). The equation uses rounding of values to 2 decimal places to increase readability.

(2) $$\left(0.92e^{\left(-0.69\cdot {\left(0.53-x_{3}\right)}^{2}-1.02\cdot {\left(0.54-x_{2}\right)}^{2}+3.96^{\left(-3.23\cdot x_{1}\right)}\right)}-0.61\right)$$

where:

$x_{1}$-vc (normalized);

$x_{2}$-g (normalized);

$x_{3}$-$f_{s}$ (normalized).

**Figure 11:**
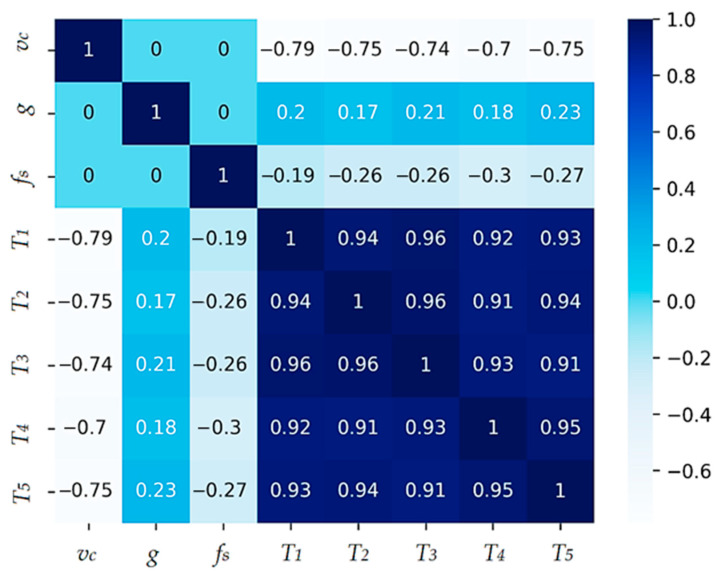
Correlation matrix.

In the case of the second model, in which the same output values are assumed, while the output values are $\left\{T_{I},T_{2},T_{3},T_{4},T_{5}\right\}$ where *T_i_*_(*i* = 1, 2, 3, 4, 5)_ equals the durability of individual tools from which the average durability value was determined, the correlation between the data was first re-examined by applying the correlation matrix. It was noticed that there is a very strong positive correlation between the output parameters. In addition, each of them correlates negatively with the parameter vc, and there is a weak correlation for the parameters g and $f_{s}$-positive and negative, respectively. A correlation matrix shows the correlation coefficients between different variables in the data set. Each element of the correlation matrix measures the degree to which two variables are related to each other. The correlation matrix is shown in Figure 11.

**Figure 12:**
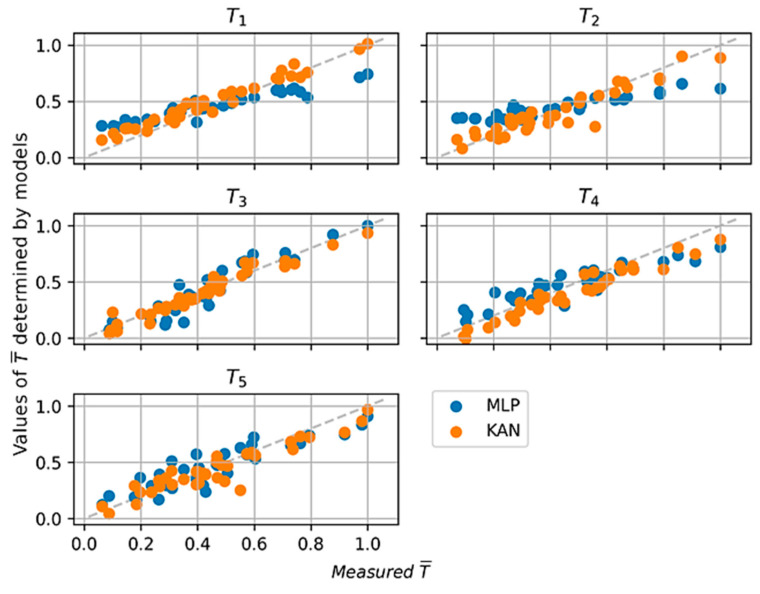
Comparison of values determined by models with measured values for MLP and KAN models.

It was decided to use the KAN model and compare it only with the Multi-Layer Perceptron (MLP) model because the other previously used models performed worse than the KAN model on every metric. Due to the multiple outputs, the RMSE-sum (RMSE) was taken as the loss function in both cases. The MLP model consists of 3 hidden layers, in the first of which the numbers of neurons are 100, 50, and 10, respectively. The ADAM (ADAptive Moment Estimation) algorithm [33] was used to optimize the model, as it allows adaptive adjustment of learning rates for each parameter. The architecture of the KAN model was changed due to the presence of multiple outputs. The number of summing nodes was assumed to be 3, and the number of multiplying nodes was also 3. The KAN model achieved a sum(RMSE) lower than the MLP model. In almost every case, the KAN model’s metrics are better than the MLP. The parameters of the KAN model were better fitted to describe the relationship between the variables due to the use of the Kolmogorov–Arnold approximation theory. Only for the output value $T_{5}$ did the MLP model achieve better values. A summary of the metrics values is shown in Table 6, and graphs comparing measured and values determined by models are shown in Figure 12.

**Figure 13:**
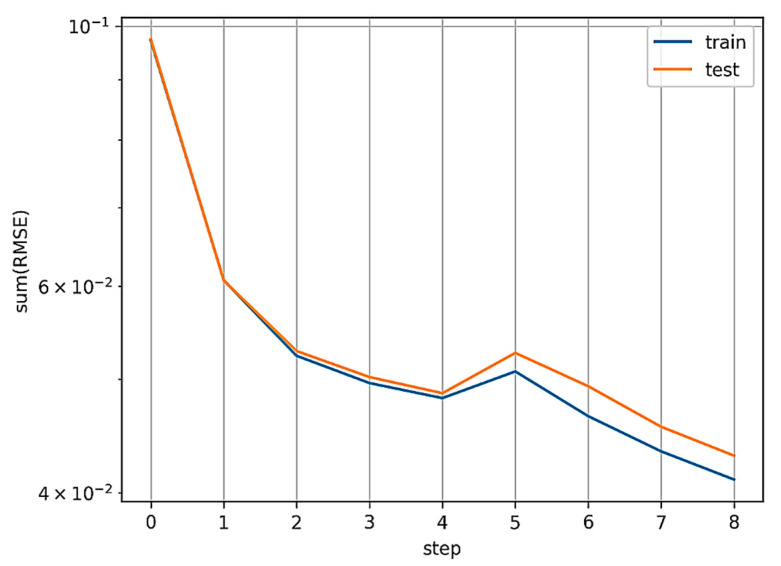
The course of the loss function values.

Based on Figure 12, it can be concluded that the KAN-based model determines values more effectively than the MLP-based model. Based on the course of the loss function values, it can be concluded that the optimal value (minimum) in the case of the KAN model was reached after step 8. The course of the value of the change in the loss function per time period is shown in Figure 13.

**Figure 14:**
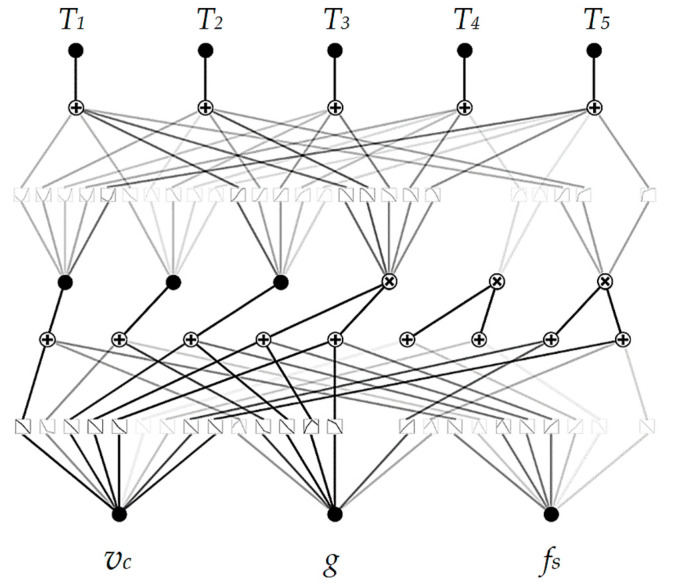
Architecture of the KAN model after simplification.

The developed KAN model retained the main connections after the pruning operation, but the less significant function approximations were removed. The least significant connection is understood to be one whose weights are close to 0 or whose impact on the final result is insignificant. The architecture of the model is shown in Figure 14.

This equation defines the model architecture shown in Figure 14. This equation is a simplification of the model architecture, which allows an accurate interpretation of the relationships between the variables that occur in the model.

This study found that using models based on Kolmogorov–Arnold approximation theory allows accurate modeling of the previously presented relationships. In addition, the operation of pruning and transformation of the model into a form described by mathematical equations increases the interpretability of the dependencies between variables described by the model. The KAN model achieved the highest metrics in the form of R^2^-score, MAE, and RMSE among the models tested.

## 5. Discussion of Results

The results of durability tests on the cutting blades of modular chisels clearly indicate that the use of titanium nitride (TiN_0.85_-Ti) coatings on cutting tools significantly improves their durability.

The tests showed that the highest tool life for cutting tools was achieved with coatings 4 µm thick (*g*_2_). As the test results and graphs in this article show, a coating that is too thin (1 µm) does not provide sufficient durability for abrasive wear, while a coating that is too thick (7 µm) may develop significant stresses during the manufacturing process. In the case of coatings produced by PVD methods that are too thick, there is a phenomenon of accumulation of residual stresses in the coating and in the transition layer between the coating and the substrate as a result of increasing defectiveness of the coating with increasing thickness. In extreme cases, too high self-stress can even lead to spontaneous coating delamination [34,35,36].

Modeling carried out as part of this study provided important insights into the durability of modular chisel blades, using both linear and non-linear models. The analysis considered the influence of three key parameters: cutting speed (*v_c_*), coating thickness (*g*), and peripheral feed (*f_s_*). The models indicated that cutting speed is negatively correlated with tool life, meaning that as cutting speed increases, blade life decreases.

A comparison of the different models showed that the Kolmogorov–Arnold Network (KAN) outperformed other models, such as Linear Regression, DecisionTreeRegressor, GradientBoostingRegressor, and AdaBoostRegressor, in terms of accuracy in describing the effect of cutting parameters on tool life. The KAN model achieved the highest R-value^2^ (0.945), which means that it was most in line with the blade life values obtained during in-service testing. An advantage of the KAN model is that it can be used to easily interpret the test results and simplify the structure of the model describing the investigated process through pruning operations. Simplifying the structure of the model enables a better understanding of the influence of individual parameters on blade life.

## 6. Conclusions

Research carried out on the application of titanium nitride coatings (TiN_0.85_-Ti) to cutting tools has shown their significant usefulness in the machining of structural steels for carburizing, particularly in the context of gear production. The main conclusions of this research are as follows:

- The most favorable coating thickness among those used was determined as follows: A coating with a thickness of 4 µm (*g*_2_) proved to be the most effective, providing the greatest cutting-edge life for the adopted blunt index value *VB_Bmax_* = 0.2 mm. Producing coatings on cutting tools that are too thick is inefficient both in terms of incurring higher manufacturing costs and in terms of the potential for significant stresses to accumulate in the coating, which can adversely affect tool life.
- The highest tool life for cutting tools was achieved with coatings 4 µm thick (*g*_2_). A coating that is too thin (1 µm) does not provide sufficient durability for abrasive wear. In the case of PVD coating methods, the too-thick (7 µm) accumulation of residual stresses. Additionally, in the transition layer between the coating and the substrate, as a result of increasing defectiveness of the coating with increasing thickness (in extreme cases), too high self-stress can even lead to spontaneous coating delamination.
- The possibility of increasing production efficiency was indicated as follows: Analyzing the obtained graphs describing the change in the durability of modular chisel blades as a function of cutting parameters, it can be assumed that the use of TiN_0.85_-Ti coatings should enable an increase in the productivity of the machining process, which is important in the context of increasing production requirements and the use of modern materials with enhanced strength properties.
- A significant effect of cutting speed on tool life was determined as follows: As mathematical modeling has shown, cutting speed (*v_c_*) negatively correlates with blade life. Optimization of this parameter is crucial to ensure long-cutting tool life.
- The advantages of the KAN model were demonstrated as follows: the Kolmogorov–Arnold Network (KAN) model proved to be the best model for describing changes in blade life as a function of machining parameters, offering high convergence with test results and the ability to easily interpret results. The modeling showed that parameters such as coating thickness and peripheral feed have a significant but variable effect with increasing values on tool life.

The TiN_0.85_-Ti coatings represent a promising technological solution for tools that are operated under impact conditions, offering, among other things, the possibility to improve the productivity and quality of gear manufacturing processes of structural steels for carburizing.

## Figures and Tables

**Table 1:** Comparison of the chemical composition and hardness of HS6-5-2 steel modular chisels with the requirements of EN ISO 4957:2002.

Specification	Chemical Composition in [%]							
	C	Mn		W	Mo	V		Cr
According to PN-86/H-85022	0.80–0.94	max 0.4		5.8–7.2	3.4–5.7	1.63–2.17		3.4–4.7
According to the analysis	0.82–0.85	0.30–0.36		6.0–6.5	4.8–5.2	1.80–2.10		4.0–4.2
**Specification**	**Chemical composition in [%]**						**HRC hardness**	
	**Si**		**P**		**S**			
According to PN-86/H-85022	max 0.5		max 0.030		max 0.030		min 64	
According to the analysis	0.20–0.30		0.020–0.025		0.018–0.020		64–65	

**Table 2:** Comparison of the chemical composition of 16HG steel with the requirements of EN 10083-1+A1:1999.

Specification	Chemical Composition in [%]						
	C	Mn	Si	Pmax.	Smax.	Cr	Ni
according to PN-EN 10083-1+A1:1999	0.14–0.19	1.00–1.30	0.17–0.37	0.035	0.035	0.80–1.10	0.30
according to the analysis	0.17	1.20	0.25	0.020	0.020	1.00	0.20

**Table 3:** Comparison of the mechanical properties of qualification specimens made of 16HG steel for gears with the requirements of EN 10083-1+A1:1999.

Specification	Strength Properties				Impact	Softened Hardness
	Rm [MPa]	Re [MPa]	As [%]	Z [%]	KC [J/cm^2^]	HB_max_
by PN-EN 10083-1+A1:1999	min 830	min 590	min 12	min 45	min 80	187
by analyzes	860 ± 20	610 ± 15	13 ± 0.3	46 ± 1.0	85 ± 2.0	180 ± 5

**Table 4:** Tool life values of modular chisel blades without and with TiN_x_-Ti coating (x = 0.85) with thicknesses *g*_1_ = 1 μm, *g*_2_ = 4 μm and *g_3_* = 7 μm as a function of peripheral feed rate *f_s_*_1_ = 0.10 mm/2·pitch, *f_s_*_2_ = 0.16 mm/2·pitch, *f_s_*_3_ =0.25 mm/2·pitch, for cutting speeds *v_c_*_1_ = 0.33 m/s, *v_c_*_1_ = 0.42 m/s, *v_c_*_1_ = 0.53 m/s, and *VB_B_*_max_ = 0.20 mm when machining serrations from 16HG steel.

g_i (i=0, 1, 2, 3)_, μm	*f_s_*_i (i=1, 2, 3)_, mm/2·pitch	*v_c_*_i (i=1, 2, 3),_ m/s	Test Number for Durability *i*					$\mathrm{Medium}\;\mathrm{Durability}\;\bar{T}$_i_ [s·10^3^]	$CI={\bar{T}}_{i}\pm z\frac{s}{\sqrt{n}}$ for *p* = 1 − α = 0.95
			1	2	3	4	5		
			Lifetime Values *T_i_*_(*i*=1, 2, 3, 4, 5)_ [s·10^3^]						
*g*_1_ = 1 μm	*f_s_*_1_ = 0.10 mm/2·pitch	*v_c_*_1_ = 0.33 m/s	51.0	56.1	52.3	61.4	60.0	56.16	56.16 ± 3.59
*g*_2_ = 4 μm			68.9	61.6	73.7	67.3	65.3	67.36	67.36 ± 3.51
*g*_3_ = 7 μm			48.0	56.1	52.2	53.8	51.9	52.40	52.40 ± 2.33
*g*_0_ = 0 μm			41.3	39.8	35.9	35.1	36.0	37.62	37.62 ± 2.16
*g*_1_ = 1 μm		*v_c_*_1_ = 0.42 m/s	35.8	35.9	33.7	40.8	39.4	37.12	37.12 ± 2.27
*g*_2_ = 4 μm			47.4	40.7	43.9	43.9	39.0	42.98	42.98 ± 2.55
*g*_3_ = 7 μm			24.9	19.3	24.7	29.4	25.8	24.82	24.82 ± 2.84
*g*_0_ = 0 μm			23.2	19.0	28.8	24.6	22.9	23.70	23.70 ± 2.77
*g*_1_ = 1 μm		*v_c_*_1_ = 0.53 m/s	17.0	23.0	21.4	19.6	20.2	20.24	20.24 ± 1.75
*g*_2_ = 4 μm			21.5	23.3	27.1	28.6	26.4	25.38	25.38 ± 2.28
*g*_3_ = 7 μm			15.3	17.0	17.1	13.8	12.9	15.22	15.22 ± 1.47
*g*_0_ = 0 μm			8.0	6.3	8.5	6.4	5.7	6.98	6.98 ± 0.94
*g*_1_ = 1 μm	*f_s_*_2_ = 0.16 mm/2·pitch	*v_c_*_1_ = 0.33 m/s	52.5	47.8	41.0	46.5	48.1	47.18	47.18 ± 3.24
*g*_2_ = 4 μm			66.9	71.3	64.7	57.2	64.0	64.82	64.82 ± 4.02
*g*_3_ = 7 μm			54.3	47.0	42.3	37.2	47.6	45.84	45.84 ± 5.01
*g*_0_ = 0 μm			26.9	33.0	32.0	38.7	32.3	32.58	32.58 ± 3.29
*g*_1_ = 1 μm		*v_c_*_1_ = 0.42 m/s	33.8	35.8	35.3	39.5	30.5	34.98	34.98 ± 2.56
*g*_2_ = 4 μm			46.7	44.8	54.7	46.7	38.6	46.30	46.30 ± 4.51
*g*_3_ = 7 μm			28.3	21.0	30.7	36.8	31.7	29.70	29.70 ± 4.52
*g*_0_ = 0 μm			20.8	15.0	19.3	23.8	17.3	19.24	19.24 ± 2.63
*g*_1_ = 1 μm		*v_c_*_1_ = 0.53 m/s	27.3	21.5	25.9	30.3	28.0	26.56	26.56 ± 2.56
*g*_2_ = 4 μm			29.0	23.8	31.8	38.0	33.0	31.20	31.20 ± 4.10
*g*_3_ = 7 μm			12.4	22.5	17.3	22.5	27.4	20.42	20.42 ± 4.49
*g*_0_ = 0 μm			10.6	15.5	6.9	12.2	12.0	11.44	11.44 ± 2.44
*g*_1_ = 1 μm	*f_s_*_3_ = 0.25 mm/2·pitch	*v_c_*_1_ = 0.33 m/s	38.2	36.2	33.6	40.5	37.5	37.20	37.20 ± 2.00
*g*_2_ = 4 μm			50.3	45.5	41.7	43.4	49.7	46.12	46.12 ± 2.98
*g*_3_ = 7 μm			29.0	32.5	35.1	35.6	30.6	32.56	32.56 ± 2.22
*g*_0_ = 0 μm			31.2	28.0	27.9	25.8	30.7	28.72	28.72 ± 1.74
*g*_1_ = 1 μm		*v_c_*_1_ = 0.42 m/s	23.5	28.2	32.4	19.8	26.1	26.00	26.00 ± 3.73
*g*_2_ = 4 μm			36.2	29.7	33.0	24.2	20.1	28.64	28.64 ± 5.11
*g*_3_ = 7 μm			21.9	9.7	14.9	17.5	25.8	17.96	17.96 ± 4.88
*g*_0_ = 0 μm			10.0	13.6	7.4	18.5	15.5	13.00	13.00 ± 3.44
*g*_1_ = 1 μm		*v_c_*_1_ = 0.53 m/s	15.6	18.5	21.1	18.2	17.2	18.12	18.12 ± 1.58
*g*_2_ = 4 μm			22.0	18.4	23.7	24.0	18.8	21.38	21.38 ± 2.08
*g*_3_ = 7 μm			7.1	9.5	8.5	7.2	11.6	8.78	8.78 ± 1.46
*g*_0_ = 0 μm			4.3	5.0	6.5	6.9	4.1	5.36	5.36 ± 1.00

**Table 5:** Comparison of analyzed models.

Model	R^2^	MAE	RMSE
Linear Regression	0.662	0.11	0.13
Decision Tree Regressor	0.894	0.06	0.07
Gradient Boosting Regressor	0.918	0.05	0.07
Ada Boost Regressor	0.924	0.05	0.06
KAN	0.945	0.04	0.05

**Table 6:** Summary of the metrics.

Model	Output	R^2^	MAE	RMSE	Sum (RMSE)
KAN	$T_{1}$	0.836	0.09	0.10	0.33
	$T_{2}$	0.884	0.06	0.08	
	$T_{3}$	0.908	0.06	0.06	
	$T_{4}$	0.827	0.08	0.09	
	$T_{5}$	0.602	0.12	0.15	
MLP	$T_{1}$	0.722	0.11	0.13	0.56
	$T_{2}$	0.573	0.11	0.15	
	$T_{3}$	0.847	0.07	0.08	
	$T_{4}$	0.793	0.09	0.10	
	$T_{5}$	0.825	0.08	0.10	

## Data Availability

The original contributions presented in this study are included in the article. Further inquiries can be directed to the corresponding author.
